# Absolute Quantification of the Host-To-Parasite DNA Ratio in *Theileria parva*-Infected Lymphocyte Cell Lines

**DOI:** 10.1371/journal.pone.0150401

**Published:** 2016-03-01

**Authors:** Hanzel T. Gotia, James B. Munro, Donald P. Knowles, Claudia A. Daubenberger, Richard P. Bishop, Joana C. Silva

**Affiliations:** 1 Institute for Genome Sciences, University of Maryland School of Medicine, 801 West Baltimore Street, Baltimore Maryland, United States of America; 2 Animal Disease Research Unit, Agricultural Research Service, US Department of Agriculture, Pullman, Washington, United States of America; 3 Department of Veterinary Microbiology & Pathology, Washington State University, Pullman, Maryland, United States of America; 4 Swiss Tropical and Public Health Institute, Socinstrasse 57, 4002 Basel, Switzerland; 5 University of Basel, Petersplatz 1, 4003 Basel, Switzerland; 6 International Livestock Research Institute, P.O. Box 30709, Nairobi 00100, Kenya; 7 Department of Microbiology and Immunology, University of Maryland School of Medicine, 685 West Baltimore Street, Baltimore Maryland, United States of America; Onderstepoort Veterinary Institute, SOUTH AFRICA

## Abstract

*Theileria parva* is a tick-transmitted intracellular apicomplexan pathogen of cattle in sub-Saharan Africa that causes East Coast fever (ECF). ECF is an acute fatal disease that kills over one million cattle annually, imposing a tremendous burden on African small-holder cattle farmers. The pathology and level of *T*. *parva* infections in its wildlife host, African buffalo (*Syncerus caffer*), and in cattle are distinct. We have developed an absolute quantification method based on quantitative PCR (qPCR) in which recombinant plasmids containing single copy genes specific to the parasite (apical membrane antigen 1 gene, *ama1*) or the host (hypoxanthine phosphoribosyltransferase 1, *hprt1*) are used as the quantification reference standards. Our study shows that *T*. *parva* and bovine cells are present in similar numbers in *T*. *parva*-infected lymphocyte cell lines and that consequently, due to its much smaller genome size, *T*. *parva* DNA comprises between 0.9% and 3% of the total DNA samples extracted from these lines. This absolute quantification assay of parasite and host genome copy number in a sample provides a simple and reliable method of assessing *T*. *parva* load in infected bovine lymphocytes, and is accurate over a wide range of host-to-parasite DNA ratios. Knowledge of the proportion of target DNA in a sample, as enabled by this method, is essential for efficient high-throughput genome sequencing applications for a variety of intracellular pathogens. This assay will also be very useful in future studies of interactions of distinct host-*T*. *parva* stocks and to fully characterize the dynamics of ECF infection in the field.

## Introduction

East Coast fever (ECF) is one of the most important livestock diseases in Africa, killing over 1 million cattle in eastern, central, and southern Africa, resulting in annual losses of over $160 million [1]. ECF is caused by the parasite *T*. *parva*, which is maintained in the African cape buffalo (*Syncerus caffer*), an asymptomatic wildlife host, and is transmitted to cattle by the ixodid tick *Rhipicephalus appendiculatus* in areas in which the two host species co-graze. Proliferation of *T*. *parva* occurs within bovine lymphocytes and susceptible animals typically die within 3 to 4 weeks post infection from severe damage to the lymphatic system and pulmonary edema [1]. Quantification of the proportion of parasite DNA in a sample containing both host and parasite DNA can be used to characterize the interactions between host and parasite, and infection dynamics within hosts and vectors [2, 3], efficacy of drug treatment [4, 5] and carrier status [6, 7]. The high-throughput DNA sequencing approach suitable for intracellular pathogens, and its success, depend on the fraction of pathogen DNA available [8, 9].

Real time PCR (qPCR) allows rapid and reliable quantification of target sequences contained within a sample [10]. Of the various methods available for quantification of nucleic acids, qPCR is a fast, sensitive, reproducible and precise technique [11]. Both conventional and qPCR are commonly used for the detection of the *T*. *parva* in lymph node biopsies or peripheral blood of infected animals but a method of simultaneous absolute quantification for both parasite and mammalian host has not yet been developed [6, 12, 13]. The qPCR techniques available for *T*. *parva* quantification measure parasite loads, allowing for comparative analyses of *T*. *parva* between ticks [2] and/or among bovine hosts [14], but are not optimal for absolute quantification. A recently described qPCR assay based on cytochrome oxidase subunit (*cox*) III is a very useful tool for the community, allowing the detection of, and discrimination among, *Theileria* species with high sensitivity. However, it is not applicable to exact quantification since *cox* III is located within the multi-copy mitochondrial genome, and may differ in amplification efficiency due to the nature and purity of the substrate [15]. Similar advantages and limitations may be posed by a qPCR assay based on 18S rDNA [16].

The biology and pathogenesis of *T*. *parva* pose considerable challenges to the generation of *T*. *parva* whole genome sequence data from a genomic DNA (gDNA) sample. Historically, *T*. *parva* genomic DNA has been isolated from the piroplasm stage [17], which requires *in vivo* infection of minimally one animal. Alternatively, purified gDNA from the macroschizont stage could be used, cultivated *in vitro* in host lymphocytes, resulting in a sample containing a mixture of bovine and parasite DNA. This raises the question of how to reliably quantify the relative abundance of host and parasite gDNA in these samples. The genome sizes differ between host and parasite, with the *T*. *parva* genome approximately 8.3 Mb [18] and the bovine genome close to 3 Gb [19]. *T*. *parva* divides in synchrony with the infected bovine lymphocytes [20], maintaining the ratio of the parasite to host DNA at a low level in cell cultures. Despite the synchronous division, estimation of the relative proportion of the DNA of the two species cannot be easily inferred even in cell culture, where it is considered likely that most bovine cells are infected. This is because the macroschizont stage of *T*. *parva* synthesizes DNA independently from the host resulting in a variable number of parasite nuclei per host cell [21]. In order to circumvent the issues associated with absolute quantification of *T*. *parva* and host DNA, we devised an absolute quantification method using qPCR in which the determination of the copy number of bovine and *T*. *parva* genes is combined with genome size information to determine the absolute amount of parasite and host DNA in a sample.

## Materials and Methods

### Samples

Four *Theileria p*arva isolates, described originally in Morzaria *et al*. [22] were used: i) Muguga: A *T*. *parva* Muguga infected lymphocyte isolate originating from an experimentally infected *Bos taurus* animal (BV115). *T*. *parva* Muguga is also the source of the parasite clone from which the *T*. *parva* reference genome was determined [23]; ii) Uganda: A *T*. *parva* isolate from North West Uganda; iii) Marikebuni: *T*. *parva* isolate from the Kenya coast; iv) *T*. *parva* buffalo 7014: *T*. *parva* buffalo (also known as *T*. *parva* lawrencei) isolated from African Cape buffalo 7014 from the northern sector of the central highlands of Kenya. The origin of all four *T*. *parva* isolates is summarized elsewhere [22]. Bovine lymphocytes infected with the schizont stage of each isolate were propagated using established protocols [24]. DNA was extracted from schizont-infected lymphocyte cell line cultures using standard protocols, incorporating proteinase K digestion, phenol/chloroform extraction, and ethanol precipitation [25].

### Ethics Statement

The Institutional Animal Care and Use Committee (IACUC) of the International Livestock Research Institute (ILRI) was established in 1993 to ensure that international standards for animal care and use are followed in all ILRI research involving use of animals. ILRI has complied voluntarily with the UK's Animals (Scientific Procedures) Act 1986 (http://www.homeoffice.gov.uk/science-research/animal-research/) that contains guidelines and codes of practice for the housing and care of animals used in scientific procedures. The study reported here was carried out in strict accordance with the recommendations in the standard operating procedures of the ILRI IACUC and adequate consideration of the 3R's (Replacement of animal with non-animal techniques, Reduction in the number of animals used, and Refinement of techniques and procedures that reduce pain and distress). Schizont-infected lymphocyte cultures were derived from lymph node biopsies taken from cattle experimentally infected with *T*. *parva* sporozoite stabilates, as described in Morzaria *et al*. [22]. The studies in which cattle were infected were specifically approved by ILRI’s IACUC. The expansion of the infected lymphocyte cultures, conducted to generate the material used in this study, does not necessitate explicit IACUC approval.

### Construction of the plasmid standards for the quantitative PCR (qPCR) assay

We constructed a plasmid standard for a parasite locus, the apical membrane antigen 1 (*ama1*) gene of *T*. *parva* from the Muguga isolate grown in animal BV115, and another for a bovine locus, the hypoxanthine phosphoribosyltransferase 1 (*hprt1*) gene amplified from gDNA from semen of a *B*. *taurus primigenius* animal, provided by the USDA, Agricultural Research Service, in Beltsville, Maryland. The primers were designed using NCBI’s primer designing tool, PRIMER-BLAST. The expected size of the PCR product was 2,089 bp for *ama1* and 1,221 bp for *hprt1* and the fragments were amplified by end point PCR. The reaction mixture of 20 μL contained the following: 10μL of *Taq* 2X Master Mix (New England BioLabs), 1μL of the 10μM forward and reverse primers, 0.6 μL of 100% DMSO, 1μL of the DNA sample solution, and 6.4μL of PCR grade water. PCR was performed using a BIO-RAD DNA Engine Thermal Cycler under the following conditions: an initial denaturation at 95°C for 15 minutes, followed by 35 cycles of 30 seconds at 95°C, 30 seconds at 60°C, and 2 minutes at 70°C. The final extension step was set for 5 minutes at 70°C.

In each case, the PCR product was purified using the QIAquick Gel Extraction Kit (Qiagen) and cloned into a TOPO TA vector (Invitrogen). The cloned plasmid was purified using QIAquick PCR purification kit (Qiagen). Inserts were confirmed with restriction digest and end point PCR (data not shown). Plasmid standards were denoted as TOPO-ama and TOPO-hprt. Plasmid constructs were individually amplified through transformation into One Shot TOP 10 chemically competent *E*. *coli* (Invitrogen), followed by overnight culture and plasmid extraction. Concentration of the plasmid standards was determined using Quant-it PicoGreen® dsDNA Assay Kit (Invitrogen).

### Sequence of *hprt1* and *ama1* fragments

In order to validate the segment for *hprt1* and *ama1* upon which the qPCR is based, amplicons were obtained from the research samples and from the bovine control DNA (*Bos taurus primigenius*) using the primers designed for qPCR. Each amplicon was purified using the QIAquick Gel Extraction Kit (Qiagen) and cloned into a TOPO TA vector (Invitrogen). The cloned plasmid was purified using QIAquick PCR purification kit (Qiagen). Plasmid constructs were individually amplified through transformation into One Shot TOP 10 chemically competent *E*. *coli* (Invitrogen), followed by overnight culture. Five individual colonies were picked for each sample, DNA extracted using the GenElute Plasmid Miniprep Kit (Sigma). For each of the five clones for each sample, the insert was amplified using the M13 Forward and M13 Reverse (M13 FR) primers sites in the vector. For each sample, each of the five replicate amplicons was sequenced directly, using Sanger sequencing, with M13 FR primers.

### qPCR assay using SYBR® Green dye

qPCR amplification and analysis were performed using Applied Biosystems 7900HT Real-Time PCR system with Sequence Detection Systems software version 2.4. The cycle of quantification (C_q_) was determined by the amplification plot in this software.

The qPCR assays were optimized to match the optimal annealing temperatures of the primer sets. The optimal conditions were 60°C for both primer sets targeting the small fragments of *ama1* and *hprt1* amplified during the qPCR assay. The qPCR mixture of 20 μL was prepared using the QuantiTect SYBR® Green PCR Kit (Qiagen): 10 μL of 2X QuantiTect SYBR® Green Master Mix, 1 μL of 10 μM forward and reverse primers, and 1 μL of template DNA. The thermal cycling protocol was set as follows: initial denaturation for 15 minutes at 95°C, followed by 45 cycles of 15 seconds at 95°C, 15 seconds at 60°C, and 15 seconds at 72°C, measuring the fluorescence signal at the end of every step. After amplification, a melting curve analysis was performed to confirm the specificity of the reaction for this sub-section. Negative controls were included in the respective reactions to confirm specificity of the primers.

### Estimation of gene copy number (GCN)

A fresh 10-fold serial dilution, ranging over seven logs of template concentration, was created from the TOPO-ama and TOPO-hprt constructs. The concentration of the plasmid preps was measured using Quant-it PicoGreen® DsDNA. The corresponding gene copy number (GCN) was calculated using the following equation, where DNA length stands for the combined length of plasmid and insert, in base pairs [26], and DNA amount equals the corresponding plasmid concentration times the volume used:

$$GCN=\frac{6.02\;\text{X}\;10^{23}\;(copy/mol)\;\text{X}\;\text{DNA amount}\;(g)}{\text{DNA length}\;(bp)\;\text{X}\;660\;(g/mol/bp)}$$ (1)

C_q_ values at each dilution were measured in triplicate using qPCR with the TOPO-ama and TOPO-hprt plasmid sets to generate the standard curves for *ama1* and *hprt1*, respectively. The C_q_ values were plotted against the logarithm of their template quantities in nanograms. Each standard curve was generated by a logarithmic regression of the plotted points. The PCR amplification efficiency, E, was calculated according the slope of each standard curve using the following equation [27]:

$$E=10^{-\frac{1}{slope}}\;-1$$

### Ratio between host and parasite DNA

The number of bovine cells was estimated as half the GCN of *hprt1*, given that they are diploid. The estimated *T*. *parva* copy number is that of *ama1*, since the parasite is haploid during its propagation in host lymphocytes. GCNs were then used to estimate the total amount of host and parasite in a sample. The total amount of DNA, in grams, given the genome size and estimated copy number, was estimated by solving the Eq (1) above for “DNA amount”. The proportion of parasite DNA in a sample was estimated by dividing the quantity of parasite DNA, in grams, by the sum of the quantity of parasite and host DNA.

## Results and Discussion

### Specificity of the qPCR reaction

**Fig 1:**
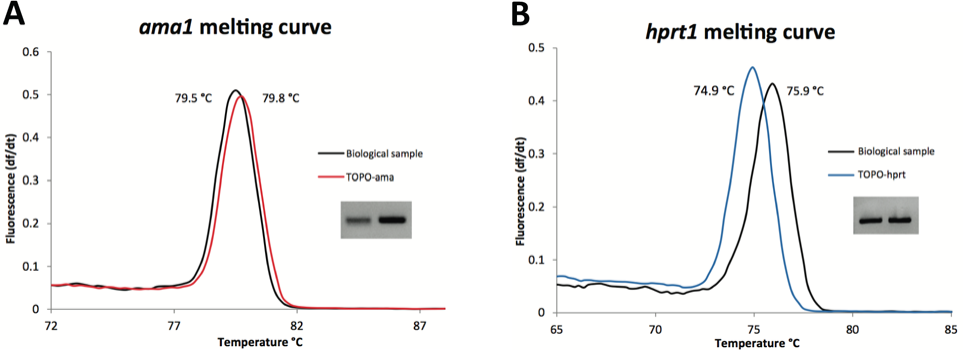
Specificity of PCR reaction in biological sample. Amplification specificity of *ama1* (**A**) and *hprt1* (**B**) was confirmed by comparing the average melting peaks for the respective primer sets using as template the plasmid DNA (colored line) or the DNA extracted from a biological sample consisting of a lymphocyte cell line, from bovine BV115, infected with the *T*. *parva* Muguga isolate (black line). Gel electrophoresis of the products was performed on a 2% agarose gel. The average peak melting temperature for *hprt1* was 74.9°C and 75.9°C, respectively, for the plasmid and the biological sample used as the source of DNA; for *ama1*, they were 79.8°C and 79.5°C, respectively for plasmid and biological sample.

Given the difficulty in obtaining pure *T*. *parva* DNA samples, free of bovine DNA, to use as quantification standards in the qPCR assay, we opted for a method involving the separate detection of parasite and host genetic markers, and their quantification in DNA samples based on standard quantification curves generated from plasmid constructs [28]. Accordingly, we generated two plasmid constructs, one that contained a fragment of the parasite gene apical membrane antigen-1 (*ama1*), amplified from the *T*. *parva* Muguga isolate [18], and the other a fragment of the bovine gene encoding hypoxanthine phosphoribosyltransferase 1 (*hprt1*), amplified from genomic DNA (gDNA) extracted from semen of a taurine bull, *Bos taurus* [29]. The *ama1* gene is commonly used for detection of piroplasm taxa (*Theileria* and *Babesia* species) both because it encodes an essential protein [30, 31], and because, unlike in *Plasmodium* [32], in piroplasms it appears to be remarkably conserved within species [33–38]. These plasmid constructs were designated TOPO-ama and TOPO-hprt, respectively. The primers used in the qPCR assay are nested within each of these fragments (Table 1). A single amplicon was obtained by this qPCR with the expected sizes of 127 and 101 base pairs (bp), respectively, for the fragments amplified from *ama1* and *hprt1* (Fig 1).

**Table 1:** Primer sequences for quantitative and conventional PCR.

Target	Accession no.	Goal	Sequence 5’ – 3’	Primer Position	Product size
***ama1***	NC_007344.1	Plasmid	F: GGAGCTAACTCTGACCCTTCG	0058–0078	2089
** **			R: CCAAAGTAGGCCAATACGGC	2128–2147	
***ama1***	NC_007344.1	qPCR	F: GCCCTTACAAGCCTTAGCTC	1726–1746	127
** **			R: GTTCGGGTGGCTTCTGGTC	1852–1834	
***hprt1***	NM_001034035	Plasmid	F: CCAGCCGGCTACGTTATGG	0022–0040	1221
** **			R: CACCAGAAATGATCTGAACAAGCA	1243–1266	
***hprt1***	NM_001034035	qPCR	F: GTTCTGTGGCCAGCTGCTTA	0714–0733	101
** **			R: AGAGTTCGGGAATGCAGCAA	0795–0814	

We compared reactions where (*i*) the plasmid preparation was used as template and (*ii*) a biological sample was used as template (Fig 1). The biological sample consisted of DNA extracted from a *T*. *parva*-infected bovine lymphocyte cell line, *T*. *parva* Muguga (Methods; [22]). The melting curve for the primer set used for *ama1* amplification peaked at nearly identical temperatures using either TOPO-ama reference plasmid or the biological sample, respectively, at 79.7°C and 79.4°C. The amplicons were 100% identical in sequence, confirming the specificity of the amplification and the conservation in this segment of the *ama1* locus (S1 Fig). The melting curves for the *hprt1* assay based on the TOPO-hprt plasmid construct and the *T*. *parva* schizont extracted DNA sample exhibited melting temperature peaks at 74.9°C and 75.9°C, respectively. The *hprt1* PCR amplicons were sequenced to confirm that the 1°C difference between the peaks in the melting curves was due to allelic differences in DNA sequence rather than to non-specific PCR amplification. We observed three single nucleotide differences between the cloned *hprt1* allele, obtained from a *Bos taurus primigeminus* animal from the United States, and the allele contained within the biological sample analyzed, BV115, which was obtained from a *Bos taurus* animal from Kenya (S1 Fig). These nucleotide differences are sufficient to account for the shift observed in the peak melting temperature [39].

### Standard quantification curves and amplification efficiency

**Fig 2:**
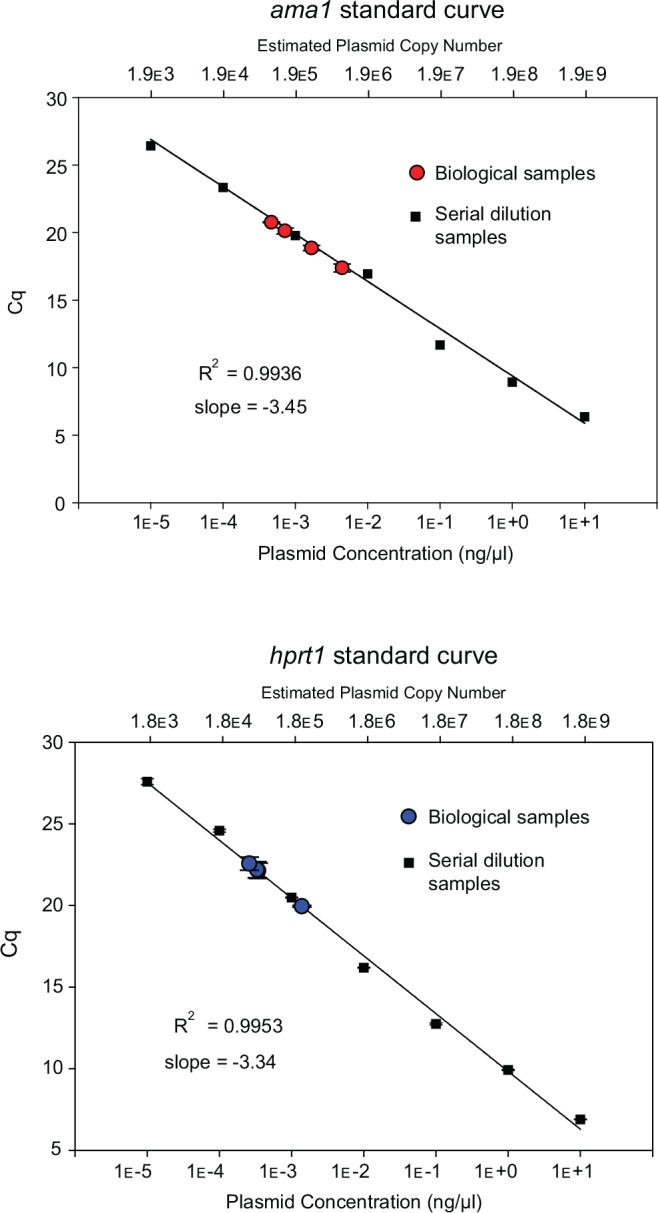
Quantification of *T*. *parva* and bovine DNA using standard reference curves for *ama1* and *hprt1*. The standard curves were constructed with seven serial 10-fold dilutions of TOPO-ama (**A**) and TOPO-hprt (**B**). Each of the plasmid dilution (n = 3 replicates per dilution; squares) and the four biological samples (*n* = 3 replicates per sample; circles) was amplified by qPCR. For each gene, the C_q_ was plotted against the logarithm of the concentration. The standard curve was generated by logarithmic regression of the average C_q_ value on the concentration of the dilutions. The original bovine and *T*. *parva* genome copy number of each biological sample was estimated by converting their respective C_q_ values into plasmid equivalents (ng/μL) using the respective regression equations and using Eq (1) in methods, to obtain the corresponding gene copy number.

The DNA concentrations of the TOPO-ama and TOPO-hprt plasmid preparations were estimated using a PicoGreen® dsDNA assay kit (Invitrogen). Aliquots were prepared from each of the plasmid preparations to initial concentrations of 1 or 10 μg/μL. The plasmid constructs were then serially diluted 10-fold and the standard quantification curves for *ama1* and *hprt1* were generated over quantities of DNA input ranging from 10 to 10^−5^ ng (Fig 2). A C_q_ (quantification cycle) value was obtained at each concentration, corresponding to the PCR cycle at which the concentration of the target DNA crossed the arbitrary threshold determined by the software (Sequence Detection Systems software version 2.4) supplied with the PCR amplification platform (Applied Biosystems 7900HT). Three replicates were run for each concentration to document reproducibility and stability of the qPCR (Table 2). The average C_q_ values were plotted against the logarithm of the respective DNA template concentrations (Fig 2). The slopes for the standard quantification curves for *ama1* and *hprt1* were -3.45 and -3.34, corresponding to relative amplification efficiencies of 94.92% and 99.25%, respectively. The R^2^ value was 0.99 in both cases.

**Table 2:** C_q_ measurements to establish standard curves.

	Concentration	C_q_1	C_q_2	C_q_3	Mean	SD
	1.00E+01	6.899761	6.9102316	6.874083	6.894692	0.01860
	1.00E+00	9.898174	9.961115	9.916024	9.925104	0.03244
	1.00E-01	12.814143	12.723901	12.686687	12.74158	0.06554
TOPO-hprt	1.00E-02	16.18242	16.16479	16.212858	16.18669	0.02432
	1.00E-03	20.515137	20.475208	20.477428	20.48926	0.02244
	1.00E-04	24.50386	24.678068	24.561707	24.58121	0.08873
	1.00E-05	27.738077	27.38037	27.623898	27.58078	0.18271
	1.00E+01	6.338894	6.364787	6.357633	6.353771	0.01337
	1.00E+00	8.946024	8.923476	8.930173	8.933224	0.01158
	1.00E-01	11.84298	11.68207	11.80879	11.77794	0.08477
TOPO-ama	1.00E-02	16.96814	16.94328	17.01416	16.97519	0.03597
	1.00E-03	19.80321	19.7777	19.87485	19.81859	0.05037
	1.00E-04	23.30352	23.34199	23.16798	23.27116	0.09141
	1.00E-05	26.55762	26.42576	26.73805	26.57381	0.15677

### Determination of sensitivity using mock infection samples

To determine the lowest level of detection and the linearity of the correlation between predicted and measured DNA, qPCR reactions were run using mixed solutions of the two plasmid constructs, in which *T*. *parva* DNA concentrations ranged from 0.5 μg/μL to 0.02 μg/μL. The accuracy was evaluated using regression analysis and resulted in R^2^ values of 0.9971 for both amplification of *ama1* and *hprt1* (S2 Fig). This represents a nearly perfect correlation between the actual and the estimated DNA concentrations.

### Ratio of *T*. *parva* to *B*. *taurus* DNA in four schizont-infected cultivated cell lines

**Fig 3:**
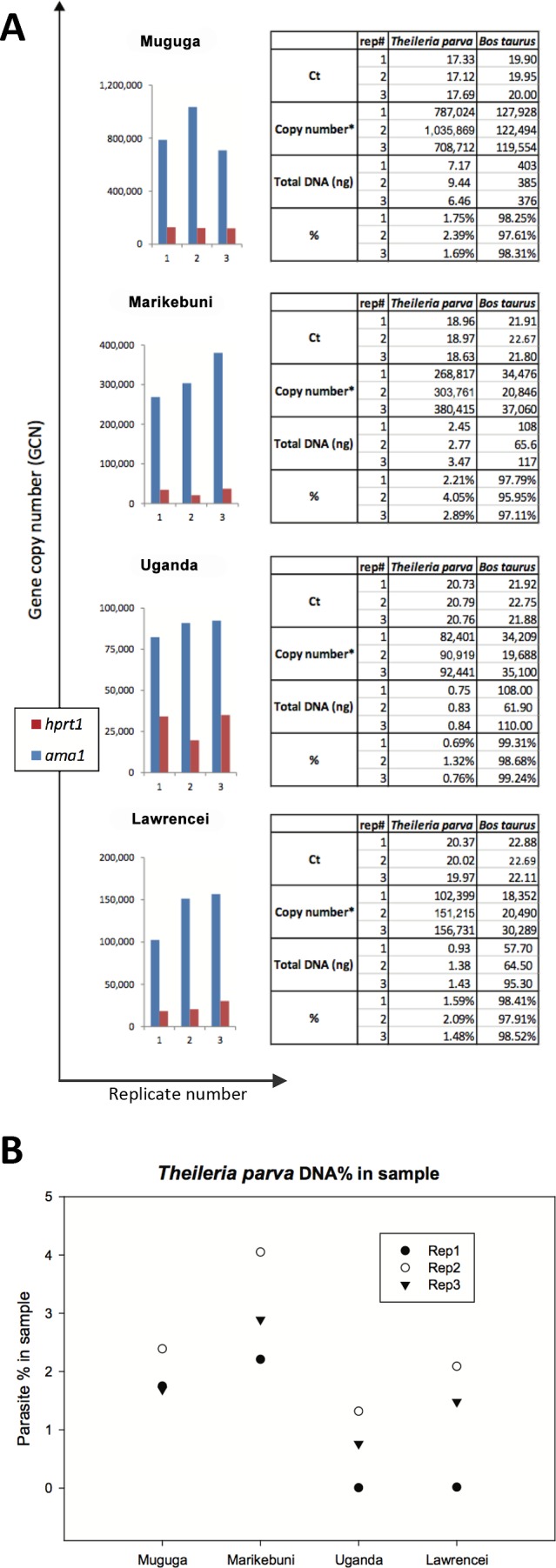
Estimation of *T*. *parva* and bovine in four biological samples. Plasmid copy numbers were inferred from C_q_ values based on the respective standard curve. Genomic copy number and the relative amount of parasite and host DNA were determined as described in Methods (A). The mean percentages are 1.94%, 3.05%, 0.92%, and 1.72% for Muguga, Marikebuni, Uganda, and buffalo 7014 (*T*. *parva* lawrencei) (B), respectively. Mean values were obtained by averaging over 3 replicates. In each case, the third qPCR replicate was done in a different day.

We quantified the amount of parasite DNA in samples of total DNA extracted from four bovine lymphocyte cell lines, each infected with a different *T*. *parva* isolate. Three of the *T*. *parva* isolates were originally obtained from cattle, namely *T*. *parva* Muguga (infected animal number BV115), *T*. *parva* Marikebuni, and *T*. *parva* Uganda. *T*. *parva* buffalo 7014 is a tissue culture isolate from a stabilate originally derived from African cape buffalo 7014, which was used to infect a *B*. *taurus* animal. The qPCR amplifications were run on dilutions of the four biological DNA samples and on the plasmid standards. Quantification of the *T*. *parva* and bovine DNA in each of the four biological samples was performed by comparing the C_q_ values for either *ama1* or *hprt1* genes with the respective standard curves generated from the plasmid construct templates, to determine the amount of plasmid equivalents (in ng per μL) using the regression equations (Fig 2). The gene copy number for each locus was then estimated by using the number of plasmid equivalents using Eq (1), described in Materials and Methods. Finally, the gene copy numbers were used to estimate the total amount of host and parasite in a sample, using Eq (1) and solving for “DNA amount”, and the proportion of *T*. *parva* DNA in the sample estimated (Fig 3).

Although the majority of the DNA is of bovine origin, *T*. *parva* parasite DNA was present in all four samples. The estimated proportion of parasite DNA in each sample was 1.94% for Muguga, 3.05% for Marikebuni, 0.92% for Uganda, and 1.72% for *T*. *parva* buffalo 7014 (Fig 3). The cell division of *T*. *parva* during its proliferation in bovine lymphocytes is synchronous with that of the host cell [13], with an estimated number of *T*. *parva* nuclei per lymphocyte ranging between 1:1 and 17:1 [20]. Since the parasite is multi-nucleated, this does not translate directly into parasite to host cell ratio. The results of the quantification approach described correspond to a ratio of *T*. *parva* to bovine nuclei between 3:1 and 10:1 for each sample. This is within the range expected for cultures in which viable, continuously proliferating host cells will necessarily contain at least one *T*. *parva* nucleus.

Previously described real time PCR assays for *T*. *parva* [2, 14, 15] were optimized for detection and to measure the relative quantity of *T*. *parva* DNA at different time points in the same infection, or between different infections. Here, we developed a novel approach based on single copy genes, each unique either to the parasite (*ama1*) or the host (*hprt1*). These two points make our assay ideal for the absolute quantification of parasite and host DNA, and for the direct comparison of those estimates, unlike other available assays. This approach will be critical for comparative analysis of *T*. *parva* infections in cattle originating from ticks that have acquired the *T*. *parva* parasite either directly from the African buffalo or from infected cattle. The former infection exhibits a low level of schizont parasitosis in conjunction with rapid host death, while the latter exhibit higher level of schizont parasitosis, but host death usually does not occur as quickly. Whether the rapid death associated with buffalo-derived parasites is related to high parasite-to-host nuclei ratios is unknown and can be addressed with this novel assay. The preliminary data presented here from a single buffalo-derived parasite 7014 infecting a bovine cell line may suggest this not to be the case. However, additional buffalo-derived parasites that have transformed bovine infected cells need to be tested before this can be confirmed.

## Conclusions

We adapted a simple, robust, accurate and precise qPCR approach to determine the ratio of *T*. *parva* to bovine DNA in a sample. The method is highly sensitive, allowing the detection of *T*. *parva* at parasite-to-host DNA ratios as low as 10^−4^ to 10^−5^. Since the quantification standard curves are based on plasmid constructs, this approach circumvents the need for pure *T*. *parva* DNA. Furthermore, this it is applicable to a wide range of research questions including, but not limited to, (*i*) the assessment of sample suitability for high-throughput sequencing, (*ii*) the evaluation of DNA extraction protocols, (*iii*) the determination of percentage schizont parasitaemia in tissue biopsies, (*iv*) monitoring the biology of in vitro cultivated bovine transformed cells lines, and (*v*) the interpretation of parasite surveillance data from the field.

## Supporting Information

S1 Fig Alignments for *ama1* and *hprt1* sequences obtained from the plasmid constructs and the biological samples. Sequence of the 127 base pair-long amplicons generated with the *ama1*-specific primers from the TOPO-ama plasmid construct and from all biological samples, aligned to the reference *T*. *parva ama1* gene (XP_766171) with Clustal W [40] (**A**). The 101 base pair PCR products generated with the *hprt1* primers were sequenced and aligned against the reference *hprt1* (NM_001034035, nucleotide positions 768–868). Allelic differences are highlighted in yellow (**B**).
(PDF)

S2 Fig Validation of quantification method using mock infection samples. Regression plot analysis of mock samples for (**A**) *ama1* and (**B**) *hprt1* were generated by incorporating various concentrations of TOPO-hprt and TOPO-ama to determine the accuracy of the standard curve.
(PDF)

## Abbreviations

ECF: East Coast fever
qPCR: quantitative PCR
*ama1*: apical membrane antigen 1
*hprt1*: hypoxanthine phosphoribosyltransferase 1
GCN: gene copy number
C_q_: cycle of quantifition

## References

[1] Norval RAI, Perry BD, Young AS. The Epidemiology of Theileriosis in Africa. London, UK: Academic Press; 1992.

[2] Odongo DO, Ueti MW, Mwaura SN, Knowles DP, Bishop RP, Scoles GA. Quantification of Theileria parva in Rhipicephalus appendiculatus (Acari: Ixodidae) confirms differences in infection between selected tick strains. Journal of medical entomology. 2009;46(4):888–94. . 19645294 10.1603/033.046.0422

[3] Medley GF, Perry BD, Young AS. Preliminary analysis of the transmission dynamics of Theileria parva in eastern Africa. Parasitology. 1993;106 (Pt 3):251–64. . 8488062 10.1017/s0031182000075077

[4] Sudarshan M, Weirather JL, Wilson ME, Sundar S. Study of parasite kinetics with antileishmanial drugs using real-time quantitative PCR in Indian visceral leishmaniasis. Journal of Antimicrobial Chemotherapy. 2011. 10.1093/jac/dkr185 PMC3133483 21609983

[5] Leang R, Barrette A, Bouth DM, Menard D, Abdur R, Duong S, et al Efficacy of Dihydroartemisinin-Piperaquine for Treatment of Uncomplicated Plasmodium falciparum and Plasmodium vivax in Cambodia, 2008 to 2010. Antimicrobial Agents and Chemotherapy. 2013;57(2):818–26. 10.1128/AAC.00686-12 23208711 PMC3553743

[6] Skilton RA, Bishop RP, Katende JM, Mwaura S, Morzaria SP. The persistence of Theileria parva infection in cattle immunized using two stocks which differ in their ability to induce a carrier state: analysis using a novel blood spot PCR assay. Parasitology. 2002;124(Pt 3):265–76. . 11922428 10.1017/s0031182001001196

[7] Bishop R, Sohanpal B, Kariuki DP, Young AS, Nene V, Baylis H, et al Detection of a carrier state in Theileria parva-infected cattle by the polymerase chain reaction. Parasitology. 1992;104 (Pt 2):215–32. . 1350672 10.1017/s0031182000061655

[8] Auburn S, Campino S, Clark TG, Djimde AA, Zongo I, Pinches R, et al An effective method to purify Plasmodium falciparum DNA directly from clinical blood samples for whole genome high-throughput sequencing. PloS one. 2011;6(7):e22213 Epub 2011/07/27. 10.1371/journal.pone.0022213 21789235 PMC3138765

[9] Takala-Harrison S, Clark TG, Jacob CG, Cummings MP, Miotto O, Dondorp AM, et al Genetic loci associated with delayed clearance of Plasmodium falciparum following artemisinin treatment in Southeast Asia. Proceedings of the National Academy of Sciences of the United States of America. 2013;110(1):240–5. Epub 2012/12/19. 10.1073/pnas.1211205110 23248304 PMC3538248

[10] Burgos JS, Ramirez C, Tenorio R, Sastre I, Bullido MJ. Influence of reagents formulation on real-time PCR parameters. Molecular and cellular probes. 2002;16(4):257–60. Epub 2002/09/25. . 12270266 10.1006/mcpr.2002.0419

[11] Ferre F. Quantitative or semi-quantitative PCR: reality versus myth. PCR methods and applications. 1992;2(1):1–9. Epub 1992/08/01. . 1490169 10.1101/gr.2.1.1

[12] Odongo DO, Sunter JD, Kiara HK, Skilton RA, Bishop RP. A nested PCR assay exhibits enhanced sensitivity for detection of Theileria parva infections in bovine blood samples from carrier animals. Parasitology research. 2010;106(2):357–65. 10.1007/s00436-009-1670-z . 19902251

[13] Bishop RP, Odongo DO, Mann DJ, Pearson TW, Sugimoto C, Haines LR, et al Theileria In: Kole C, Nene V, editors. Genome Mapping in Animal Associated Microbes. Berlin-Heidelberg: Springer-Verlag; 2009 p. 191–231.

[14] Yamada S, Konnai S, Imamura S, Simuunza M, Chembensofu M, Chota A, et al PCR-based detection of blood parasites in cattle and adult Rhipicephalus appendiculatus ticks. Vet J. 2009;182(2):352–5. 10.1016/j.tvjl.2008.06.007 . 18700182

[15] Chaisi ME, Janssens ME, Vermeiren L, Oosthuizen MC, Collins NE, Geysen D. Evaluation of a real-time PCR test for the detection and discrimination of theileria species in the African buffalo (Syncerus caffer). PloS one. 2013;8(10):e75827 10.1371/journal.pone.0075827 24146782 PMC3798391

[16] Sibeko KP, Oosthuizen MC, Collins NE, Geysen D, Rambritch NE, Latif AA, et al Development and evaluation of a real-time polymerase chain reaction test for the detection of Theileria parva infections in Cape buffalo (Syncerus caffer) and cattle. Vet Parasitol. 2008;155(1–2):37–48. 10.1016/j.vetpar.2008.03.033 . 18514421

[17] Conrad PA, Iams K, Brown WC, Sohanpal B, ole-MoiYoi OK. DNA probes detect genomic diversity in Theileria parva stocks. Mol Biochem Parasitol. 1987;25(3):213–26. . 2892129 10.1016/0166-6851(87)90085-5

[18] Gardner MJ, Bishop R, Shah T, de Villiers EP, Carlton JM, Hall N, et al Genome sequence of Theileria parva, a bovine pathogen that transforms lymphocytes. Science. 2005;309(5731):134–7. Epub 2005/07/05. 10.1126/science.1110439 . 15994558

[19] Elsik CG, Tellam RL, Worley KC, Gibbs RA, Muzny DM, Weinstock GM, et al The genome sequence of taurine cattle: a window to ruminant biology and evolution. Science. 2009;324(5926):522–8. Epub 2009/04/25. 10.1126/science.1169588 19390049 PMC2943200

[20] Hulliger L, Wilde JKH, Brown CGD, Turner L. Mode of multiplication of Theileria in cultures of bovine lymphocyte cells. Nature. 1964;203:728–30. 10.1038/203728a0 14207267

[21] Irvin AD, Ocama J, Spooner P. Cycle of bovine lymphoblastoid cells parasitised by Theileria parva. Research in Veterinary Science. 1982;33(3):298–304. 6818647

[22] Morzaria SP, Dolan TT, Norval RAI, Bishop RP, Spooner PR. Generation and characterization of cloned Theileria parva parasites. Parasitology. 1995;111(01):39–49. 7609989 10.1017/s0031182000064581

[23] Gardner MJ, Bishop R, Shah T, de Villiers EP, Carlton JM, Hall N, et al Genome sequence of Theileria parva, a bovine pathogen that transforms lymphocytes. Science. 2005;309(5731):134–7. 10.1126/science.1110439 . 15994558

[24] Brown CGD. Propagation of *Theileria* In: Maramorosch K, Hirumi H, editors. Practical Tissue Culture Applications: Academic Press; 1979 p. 223–45.

[25] Sambrook J, Fitsch E, Maniatis T. Molecular Cloning: A Laboratory Manual. Cold Spring Harbor: Cold Spring Harbor Press; 1989.

[26] Whelan JA, Russell NB, Whelan MA. A method for the absolute quantification of cDNA using real-time PCR. Journal of immunological methods. 2003;278(1–2):261–9. Epub 2003/09/06. . 12957413 10.1016/s0022-1759(03)00223-0

[27] Rasmussen R. Quantifiation on the LightCycler In: Meuer S, Wittwer C, Nakagawara K-I, editors. Rapid Cycle Real-Time PCR. Berlin: Springer-Verlag; 2001 p. 21–34.

[28] Lee C, Kim J, Shin SG, Hwang S. Absolute and relative QPCR quantification of plasmid copy number in Escherichia coli. Journal of biotechnology. 2006;123(3):273–80. Epub 2006/01/04. 10.1016/j.jbiotec.2005.11.014 . 16388869

[29] Goossens K, Van Poucke M, Van Soom A, Vandesompele J, Van Zeveren A, Peelman LJ. Selection of reference genes for quantitative real-time PCR in bovine preimplantation embryos. BMC Dev Biol. 2005;5:27 10.1186/1471-213X-5-27 16324220 PMC1315359

[30] Besteiro S, Dubremetz JF, Lebrun M. The moving junction of apicomplexan parasites: a key structure for invasion. Cell Microbiol. 2011;13(6):797–805. 10.1111/j.1462-5822.2011.01597.x . 21535344

[31] Harvey KL, Yap A, Gilson PR, Cowman AF, Crabb BS. Insights and controversies into the role of the key apicomplexan invasion ligand, Apical Membrane Antigen 1. Int J Parasitol. 2014;44(12):853–7. 10.1016/j.ijpara.2014.08.001 . 25157917

[32] Takala SL, Coulibaly D, Thera MA, Batchelor AH, Cummings MP, Escalante AA, et al Extreme polymorphism in a vaccine antigen and risk of clinical malaria: implications for vaccine development. Sci Transl Med. 2009;1(2):2ra5 10.1126/scitranslmed.3000257 20165550 PMC2822345

[33] Elsify A, Sivakumar T, Nayel M, Salama A, Elkhtam A, Rizk M, et al An epidemiological survey of bovine Babesia and Theileria parasites in cattle, buffaloes, and sheep in Egypt. Parasitol Int. 2015;64(1):79–85. 10.1016/j.parint.2014.10.002 . 25305419

[34] Moreau E, Bonsergent C, Al Dybiat I, Gonzalez LM, Lobo CA, Montero E, et al Babesia divergens apical membrane antigen-1 (BdAMA-1): A poorly polymorphic protein that induces a weak and late immune response. Exp Parasitol. 2015;155:40–5. 10.1016/j.exppara.2015.04.024 . 25956948

[35] Sivakumar T, Altangerel K, Battsetseg B, Battur B, Aboulaila M, Munkhjargal T, et al Genetic detection of Babesia bigemina from Mongolian cattle using apical membrane antigen-1 gene-based PCR assay. Vet Parasitol. 2012;187(1–2):17–22. 10.1016/j.vetpar.2012.01.008 . 22284301

[36] Sivakumar T, Tattiyapong M, Fukushi S, Hayashida K, Kothalawala H, Silva SS, et al Genetic characterization of Babesia and Theileria parasites in water buffaloes in Sri Lanka. Vet Parasitol. 2014;200(1–2):24–30. 10.1016/j.vetpar.2013.11.029 . 24365246

[37] Torina A, Agnone A, Sireci G, Mosqueda JJ, Blanda V, Albanese I, et al Characterization of the apical membrane antigen-1 in Italian strains of Babesia bigemina. Transbound Emerg Dis. 2010;57(1–2):52–6. 10.1111/j.1865-1682.2010.01118.x . 20537104

[38] Yoshinari T, Sivakumar T, Asada M, Battsetseg B, Huang X, Lan DT, et al A PCR based survey of Babesia ovata in cattle from various Asian, African and South American countries. J Vet Med Sci. 2013;75(2):211–4. . 23037864 10.1292/jvms.12-0329

[39] Kibbe WA. OligoCalc: an online oligonucleotide properties calculator. Nucleic acids research. 2007;35(Web Server issue):W43–6. 10.1093/nar/gkm234 17452344 PMC1933198

[40] Thompson JD, Higgins DG, Gibson TJ. CLUSTAL W: improving the sensitivity of progressive multiple sequence alignment through sequence weighting, position-specific gap penalties and weight matrix choice. Nucleic acids research. 1994;22(22):4673–80. 7984417 10.1093/nar/22.22.4673 PMC308517

