# Supplementary material for: Absolute Quantification of the Host-To-Parasite DNA Ratio in Theileria parva-Infected Lymphocyte Cell Lines
Source: PLoS One. 2016 Mar 1;11(3):e0150401. doi: 10.1371/journal.pone.0150401 (PMC4773007; doi:10.1371/journal.pone.0150401)
Supplement: S1 Fig — Sequence of the 127 base pair-long amplicons generated with the ama1-specific primers from the TOPO-ama plasmid construct and from all biological samples, aligned to the reference T. parva ama1 gene (XP_766171) with Clustal W [40] (A). The 101 base pair PCR products generated with the hprt1 primers were sequenced and aligned against the reference hprt1 (NM_001034035, nucleotide positions 768–868). Allelic differences are highlighted in yellow (B). (PDF) [file pone.0150401.s001.pdf]

```
A
                  Fwd. primer                                                                                    Rev. primer
XP_766171     GGGCCACATACTCTGTCGAAGAAAAGAAATGTAACATTCTCGACGTTGTTCCCACCTGCCTTATTATAAGTAACGGCTACTACGCCCTTACAAGCCTTAGCT
TOPO-ama      GGGCCACATACTCTGTCGAAGAAAAGAAATGTAACATTCTCGACGTTGTTCCCACCTGCCTTATTATAAGTAACGGCTACTACGCCCTTACAAGCCTTAGCT
BV115         GGGCCACATACTCTGTCGAAGAAAAGAAATGTAACATTCTCGACGTTGTTCCCACCTGCCTTATTATAAGTAACGGCTACTACGCCCTTACAAGCCTTAGCT
Marikebuni    GGGCCACATACTCTGTCGAAGAAAAGAAATGTAACATTCTCGACGTTGTTCCCACCTGCCTTATTATAAGTAACGGCTACTACGCCCTTACAAGCCTTAGCT
Uganda        GGGCCACATACTCTGTCGAAGAAAAGAAATGTAACATTCTCGACGTTGTTCCCACCTGCCTTATTATAAGTAACGGCTACTACGCCCTTACAAGCCTTAGCT
Lawrencei     GGGCCACATACTCTGTCGAAGAAAAGAAATGTAACATTCTCGACGTTGTTCCCACCTGCCTTATTATAAGTAACGGCTACTACGCCCTTACAAGCCTTAGCT
              ******************************************************************************************************

B
                  Fwd. primer                                                                                   Rev. primer
NM_001034035  AGAGTTCGGGAATGCAGCAACTGACATTTCTAAAATACAAAACAGATCAAATTCTTAGGAGATACATGCAATAAGCTCTATTAAGCAGCTGGCCACAGAAC
TOPO-hprt     AGAGTTCGGGAATGCAGCAACTGACATTTCTAAAATACAAAACAGATCAAATTCTTAGGAGATACATGCAATAAGCTCTATTAAGCAGCTGGCCACAGAAC
BV115         AGAGTTCGGGAATGCAGCAACTGACATTTCTAAAGTACAAAACAGATCAAATTCTTAGGAGAGACATGCAGTAAGCTCTATTAAGCAGCTGGCCACAGAAC
              ********************************** *************************** ******* ******************************
```
