# Supplementary figures and images for: Absolute Quantification of the Host-To-Parasite DNA Ratio in Theileria parva-Infected Lymphocyte Cell Lines
Source: PLoS One. 2016 Mar 1;11(3):e0150401. doi: 10.1371/journal.pone.0150401 (PMC4773007; doi:10.1371/journal.pone.0150401)

**A**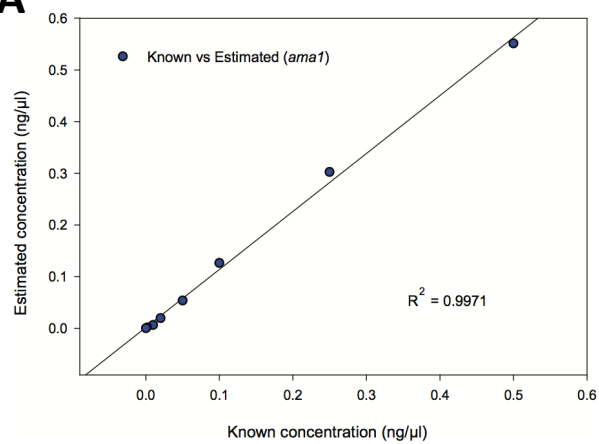**B**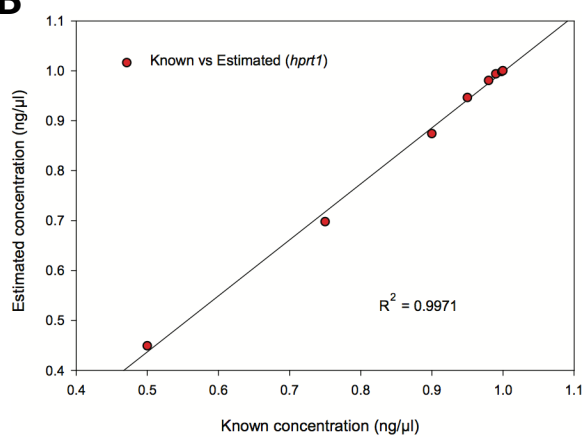

Supplement: S2 Fig — Regression plot analysis of mock samples for (A) ama1 and (B) hprt1 were generated by incorporating various concentrations of TOPO-hprt and TOPO-ama to determine the accuracy of the standard curve. (PDF) [file pone.0150401.s002.pdf]
